# Pool boiling of water-Al_2_O_3 _and water-Cu nanofluids on horizontal smooth tubes

**DOI:** 10.1186/1556-276X-6-220

**Published:** 2011-03-15

**Authors:** Janusz T Cieslinski, Tomasz Z Kaczmarczyk

**Affiliations:** 1 Department of Ecoengineering and Process Apparatus, Gdansk University of Technology, Narutowicza 11/12, 80-233 Gdansk, Poland

## Abstract

Experimental investigation of heat transfer during pool boiling of two nanofluids, i.e., water-Al_2_O_3 _and water-Cu has been carried out. Nanoparticles were tested at the concentration of 0.01%, 0.1%, and 1% by weight. The horizontal smooth copper and stainless steel tubes having 10 mm OD and 0.6 mm wall thickness formed test heater. The experiments have been performed to establish the influence of nanofluids concentration as well as tube surface material on heat transfer characteristics at atmospheric pressure. The results indicate that independent of concentration nanoparticle material (Al_2_O_3 _and Cu) has almost no influence on heat transfer coefficient while boiling of water-Al_2_O_3 _or water-Cu nanofluids on smooth copper tube. It seems that heater material did not affect the boiling heat transfer in 0.1 wt.% water-Cu nanofluid, nevertheless independent of concentration, distinctly higher heat transfer coefficient was recorded for stainless steel tube than for copper tube for the same heat flux density.

## Introduction

Recent advances in nanotechnology have allowed development of a new category of liquids termed nanofluids, which was first used by a group in Argonne National Laboratory USA [1] to describe liquid suspensions containing nanoparticles with thermal conductivities, orders of magnitudes higher than the base liquids, and with sizes significantly smaller than 100 nm. The augment of thermal conductivity could provide a basis for an enormous innovation for heat transfer intensification, which is pertinent to a number of industrial sectors including transportation, power generation, micro-manufacturing, chemical and metallurgical industries, as well as heating, cooling, ventilation, and air-conditioning industry. Literature findings regarding pool boiling of nanofluids can be summarized as follows.

Li et al. [2] studied boiling of water-CuO nanofluids of different concentrations (0.05% and 0.2% by weight) on copper plate. They observed deterioration of heat transfer as compared to the base fluid and attributed this fact to the sedimentation of nanoparticles which leads to the changing of radius of cavity, contact angle, and superheat layer thickness.

You et al. [3] reported that independent of the concentration of the nanoparticles (0.001 to 0.05 g/l) nucleate boiling heat transfer coefficients for water-Al_2_O_3 _nanofluid while boiling on plate appeared to be the same as for base fluid. They also found that the size of bubbles increased with addition of nanoparticles to water.

Das et al. [4] conducted an investigation on the pool boiling of water-Al_2_O_3 _nanofluids on a horizontal tubular heater having a diameter of 20 mm with different surface roughness at atmospheric pressure. It was found that the boiling heat transfer of nanoparticle-suspensions was deteriorated compared to that of pure water. Compared with pure water, surface roughness of the heating surface could also greatly affect the nucleation superheat. The subsidence of nanoparticles was considered as the main reason for the increase of the superheat.

Vassallo et al. [5] carried out an experiment of water-SiO_2 _nanofluids boiling on a horizontal NiCr wire at atmospheric pressure. No appreciable differences in the boiling heat transfer were found for the heat flux less than the CHF.

Bang and Chang [6] conducted an experimental investigation on the pool boiling of water-Al_2_O_3 _nanofluids on a plain plate at atmospheric pressure. The concentration of nanoparticles was 0.5%, 1%, 2%, and 4% by volume. It was found that the boiling curves were shifted right - towards higher wall superheats. The deterioration became worse as nanoparticle concentration increased and was related to the change of the heating surface characteristics by the deposition of nanoparticles on the heating surface.

Wen and Ding [7] studied boiling of water-Al_2_O_3 _nanofluids on a stainless steel disc with 150 mm in diameter at atmospheric pressure. Contrary to the Bang and Chang's work [6], heat transfer enhancement has been recorded. Possible explanation of this controversy is lower concentration of nanoparticles used (0.32%).

Shi et al. [8] carried out experiments with boiling of water-Al_2_O_3 _nanofluid and Fe-water nanofluid on horizontal, copper plate with 60 mm in diameter. The concentration of nanoparticles was 0.1%, 1%, and 2% by volume. Generally, the augmentation and deterioration of heat transfer was observed for water-Fe and water-Al_2_O_3 _nanofluids, respectively.

Nguyen et al. [9] investigated boiling of water-Al_2_O_3 _nanofluid on chrome-plated, very smooth face of copper block of a 100 mm diameter. The concentration of nanoparticles was 0.5%, 1%, and 2% by volume. In general, it was observed that for a given wall superheat, the heat flux considerably decreased with the increase of the particle concentration. Furthermore, for sufficiently high wall superheat, the heat flux tended to become nearly constant.

Coursey and Kim J. [10] showed that even if the Al_2_O_3 _nanoparticle concentration was increased by over two orders of magnitude, no enhancement or degradation of heat transfer was observed during boiling of ethanol-based nanofluids on glass or gold surface. It was attributed to the highly wetting nature of ethanol. For ethanol-Al_2_O_3 _nanofluids and copper surfaces, the nucleate boiling was improved with increasing nanoparticle concentration.

Liu and Liao [11] examined nanofluids, i.e., mixture of base fluid (water and alcohol), the nanoparticles (CuO and SiO_2_) and the surfactant (SDBS), and nanoparticles-suspensions consisted of the base liquid and nanoparticles during pool boiling on the face of copper bar having 20 mm diameter. The boiling characteristics of the nanofluids and nanoparticles- suspensions are poorer compared with that of the base fluids.

Narayan et al. [12] studied influence of tube orientation on pool boiling heat transfer of water-Al_2_O_3 _nanofluids from a smooth tube of diameter 33 mm inclined at 0°, 45°, and 90°. They found that horizontal orientation gave maximum heat transfer and the boiling performance deteriorated with increase in nanoparticle concentration (0.25%, 1%, and 2% by weight).

Lotfi and Shafii [13] performed transient quenching experiments with silver sphere 10 mm diameter immersed in water-Ag and water-TiO_2 _nanofluids. It was established that the quenching process was more rapid in pure water than in nanofluids and the cooling time was inversely proportional to the nanoparticle mass concentration (0.5%, 1%, 2%, and 4% - Al_2_O_3 _and 0.125%, 0.255, 0.5%, and 1% - TiO_2_).

Trisaksri and Wongwises [14] tested R141b-TiO_2 _nanofluids while boiling on horizontal copper cylinder 28.5 mm diameter. They discovered that adding a small amount of nanoparticles did not affect the boiling heat transfer, but addition of TiO_2 _nanoparticles at 0.03% and 0.05% by volume deteriorated the boiling heat transfer. Moreover, the boiling heat transfer coefficient decreased with increasing particle volume concentrations, especially at higher heat flux.

Kathiravan et al. [15] investigated boiling of water-Cu and water-Cu-SDS (9 wt.%) nanofluids on a 300 mm square stainless steel plate. They revealed that copper nanoparticles caused a decrease in boiling heat transfer coefficient for water as base liquid. The heat transfer coefficient decreased with increase of the concentration of nanoparticles (0.25%, 0.5%, and 1% by weight) for both water-Cu and water-Cu-SDS nanofluids.

Suriyawong and Wongwises [16] studied boiling of water-TiO_2 _nanofluids on horizontal circular plates made from copper and aluminium with different roughness (0.2 and 4 μm). The concentration of nanoparticles was very low: 0.00005%, 0.0001%, 0.0005%, 0.005%, and 0.01% by volume. For copper plate with nanofluid's concentrations more than 0.0001%, the heat transfer coefficient was found to be less than that of the base fluid at both levels of surface roughness. On the other hand, for aluminium surfaces the heat transfer coefficient was found to be less than that of base fluid at every level of nanofluids concentration and surface roughness.

Ahmed and Hamed [17] performed experiments with boiling of water-Al_2_O_3 _on a face of copper block of 25.4 mm diameter. Nanofluids at 0.01%, 0.1%, and 0.5% by volume concentrations were prepared at a neutral pH of 6.5 and an acidic pH of 5. Ultrasonic vibration and electrostatic stabilization were used to prepare nanofluids. It was found that concentration increase either reduced or had no effect on heat transfer coefficient. Enhancement of heat transfer coefficient was achieved only at low nanofluid concentration (0.01%) and the nanofluid at a pH of 6.5.

Recently, Kwark et al. [18] pointed out the transient characteristics of water-Al_2_O_3 _nanofluid boiling on horizontal copper plate. The longer a heater is subjected to nanofluid boiling process, the thicker the nanoparticle coating generated on its surface. The thickness of this nanoparticle coating can then dictate boiling heat transfer coefficient.

The currently available experimental data on boiling heat transfer of nanofluids are still limited. Additionally, conflicting results as far as effect of nanoparticles on the pool boiling heat transfer performance have been reported [19,20]. As suggested in [21], further detailed investigations are necessary to understand the phenomena of boiling of nanofluids. In particular experiments are lacking on the effects of nanoparticles material and heating surface material on boiling heat transfer from horizontal smooth tubes. As a consequence, the main aim of the present study was to obtain boiling characteristics, i.e., boiling curves and heat transfer coefficients for water-Al_2_O_3 _and water-Cu nanofluids of different concentrations for copper and stainless steel tubes.

## Experimental

### Experimental set-up

**Figure 1:**
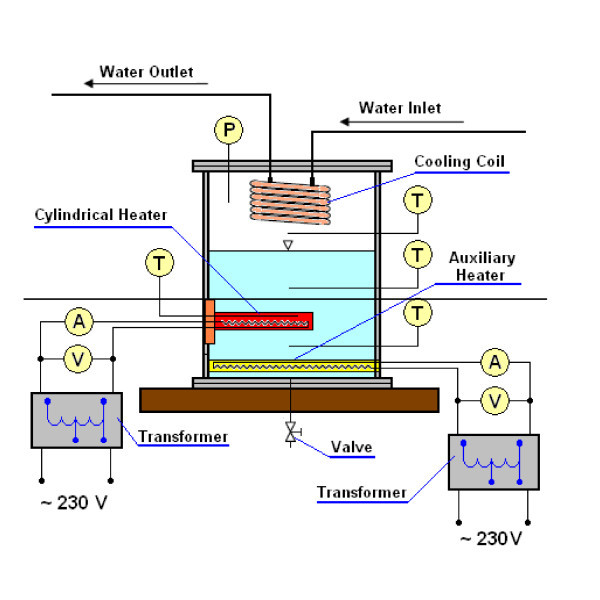
**Scheme of the experimental rig**.

**Figure 2:**
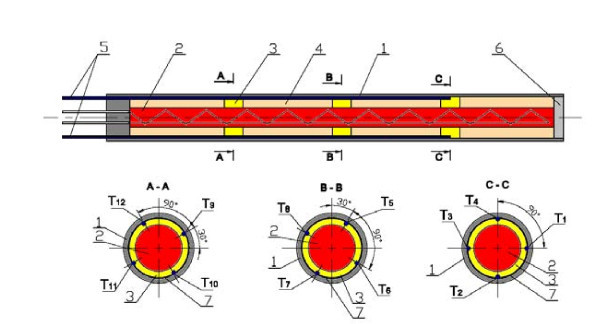
**Details of the test section**. 1 - heating surface, 2 - cartridge heater, 3 - Teflon ring, 4 - copper sleeve, 5 - thermocouples, 6 - insulating cap, 7 - thermoconductive paste.

Figure 1 shows a schematic diagram of the experimental apparatus. The test chamber consisted of a cubical vessel made of stainless steel with inside dimensions of 150 × 150 × 250 mm. The horizontal copper and stainless steel tubes having 10 mm OD and 0.6 mm wall thickness formed test heater. The effective length of the test tube was 100 mm. The stainless steel tube was finished with emery paper 400 (*R*_a _= 0.08 μm) and copper tube was polished with abrasive compound (R_a _= 0.12 μm). The test tube was cantilever-mounted from the back wall of the test chamber to permit visualization. A resistance cartridge heater was inserted into the test tube to generate heat flux from an electrical power supply. The power supply was adjusted by an electrical transformer. Great care must be exercised with the cartridge heater and temperature measuring instrumentation to ensure good accuracy of the measurement of the inside temperature of the heating cylinder [22]. In the present study, inside the tube, a copper sleeve with 12 grooves at the outside surface to locate the thermocouples was inserted. The copper sleeve was divided into three equal parts, which were separated by three Teflon, 4 mm long, rings. Twelve K-type thermocouples for surface temperature measurements were installed in the grooves. The ends of the thermocouples were placed in the grooves in the middle of the Teflon ring in order to avoid the influence of the heater on the reading from the thermocouples. The detailed geometry of the test tube is shown in Figure 2. The wall temperature *t*_w _was calculated from the formula [23](1)

where *U *and *I *are cartridge heater voltage drop and current, respectively, *D_o_/D_i _*is the outside to inside diameter ratio, *L *is an active length of a tube, *λ *is a thermal conductivity of a tube material (copper or stainless steel) and *t_i _*was calculated as the arithmetic mean of 12 measured inside wall temperatures. The liquid level was maintained at ca. 15 mm above the centerline of the test tube at saturated state.

### Preparation and characterization of the tested nanofluids

**Figure 3:**
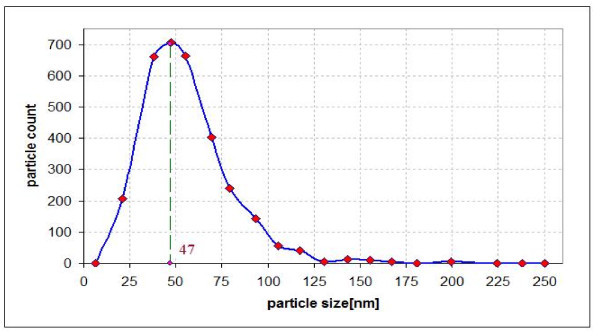
**Particle size distribution of the Al_2_O_3 _nano-powder**.

**Figure 4:**
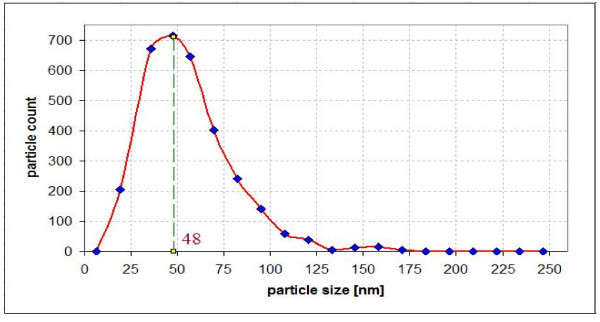
**Particle size distribution of the Cu nano-powder**.

**Figure 5:**
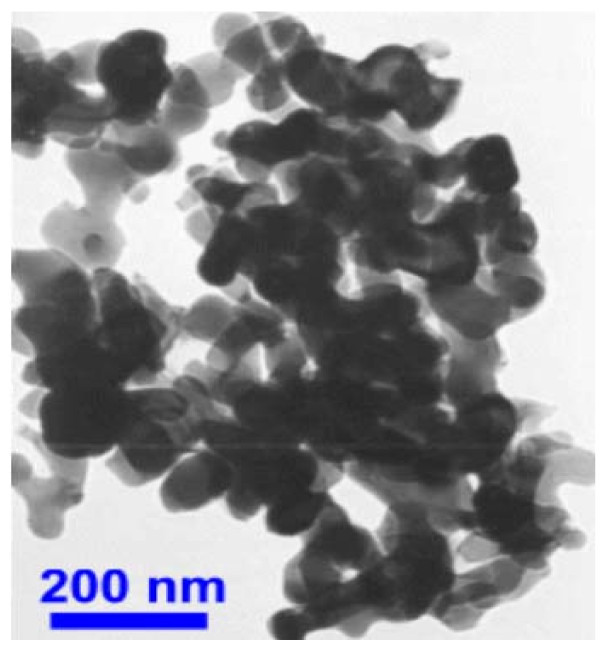
**TEM of agglomerated nano-aluminium oxide powder**.

**Figure 6:**
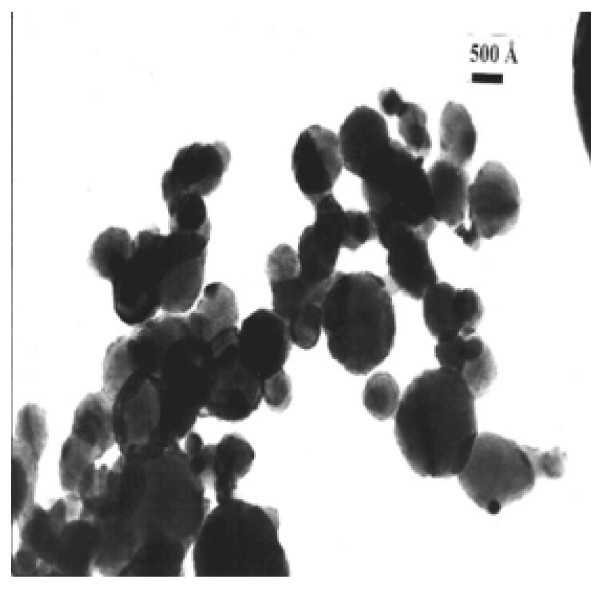
**TEM of agglomerated nano-copper powder**.

In the present study, Al_2_O_3 _an Cu were used as nanoparticles while distilled, deionized water was used as a base fluid. Nanofluids with different concentrations were prepared for the experiments. Nanoparticles of the required amount and base fluid were mixed together. Alumina (Al_2_O_3_) nanoparticles, of spherical form have diameter from 5 to 250 nm; their mean diameter was estimated to be 47 nm according to the deliverer (Sigma-Aldrich Co., Poznan, Poland). Copper nanoparticles, of spherical form have diameter from 7 to 257 nm; their mean diameter was estimated to be 48 nm according to the deliverer (Sigma-Aldrich Co., Poznan, Poland). The alumina and copper particle size distributions are shown in Figures 3 and 4, respectively. In the powder state, the nanoparticles form loose agglomerates of micrometer size as shown by transmission electron microscopy (TEM) - Figures 5 and 6. However, it has been observed that the agglomerates breakdown to a considerable extent to produce smaller size particles and agglomerates when dispersed in water.

Dispersants were not used to stabilize the suspension. Ultrasonic vibration was used for 4-5 h in order to stabilize the dispersion of the nanoparticles. Nanoparticles were tested at the concentration of 0.01%, 0.1%, and 1% by weight.

**Figure 7:**
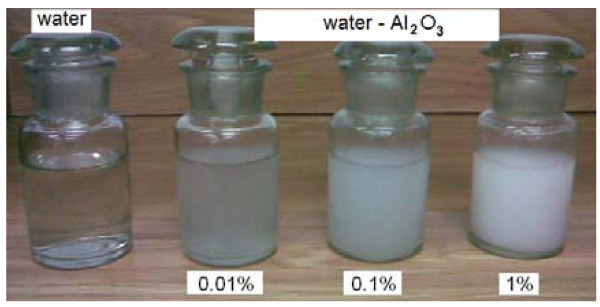
**Photographs of the water-Al_2_O_3 _nanofluids**.

**Figure 8:**
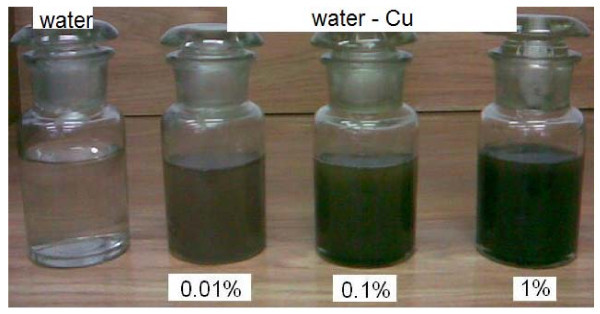
**Photographs of the water-Cu nanofluids**.

Figures 7 and 8 display photographs of the tested water-Al_2_O_3 _and water-Cu nanofluids, respectively.

**Figure 9:**
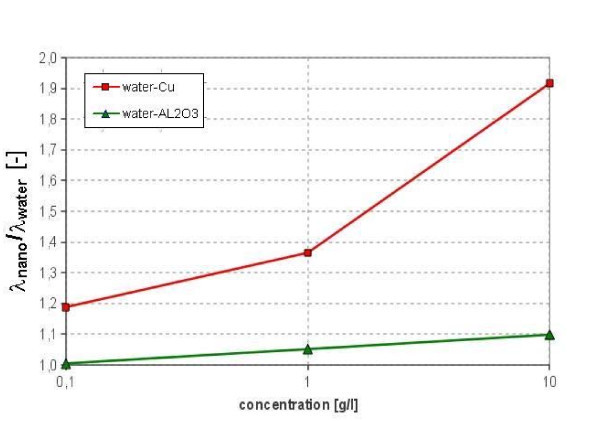
**Effective thermal conductivity of water-Al_2_O_3 _and water-Cu nanofluids at ambient temperature and various concentrations**.

The thermal conductivity of the nanofluids was measured using transient hot-wire method (KD2 Pro by Decagon Device Inc.). The results for measurements at ambient temperature range between 18 and 20°C are shown in Figure 9. Each data point is an average value of six measurements and the measurement error ranges within ± 5%. It can be seen in Figure 9 that the effective thermal conductivity of nanofluids increases considerably against mass concentration, and an enhancement of approximately 10% and 90% are achieved at a particle concentration of 1% by weight for water-Al_2_O_3 _and water-Cu nanofluids, respectively. Reasonable agreement between present data and Wen and Ding [7] results for water-Al_2_O_3 _nanofluids can be observed. It is difficult to compare present data of thermal conductivity of water-Cu nanofluids with selected published results because of big scatter of literature data [24].

### Experimental procedure

In a typical experiment, before the test begins, a vacuum pump was used to evacuate the accumulated air from the vessel. Nanofluid at a preset concentration was charged and then preheated to the saturated temperature by auxiliary heater. Next, the cartridge heater was switched on. Measurement was first performed at the lowest power input. Data were collected by increasing the heat flux by small increments. Experiments were performed at atmospheric pressure. Each data point was taken at steady state, the condition of steady state being defined as a variation in the thermocouple outputs of less than 0.001 mV during the 3 min. It generally took about 15 min to achieve steady conditions after the power level was changed.

In order to ensure consistent surface state after each test, the boiling surface was prepared in the same manner, i.e., the stainless steel tube was finished with emery paper 400 and copper tube was polished with abrasive compound, next the test tube was placed in an ultrasonic cleaner for 1 h. Finally, the boiling surface was cleaned by water jet.

### Uncertainty estimation

The uncertainties of the measured and calculated parameters are estimated by mean-square method. Because heat flux density was calculated from the formula(2)

the experimental uncertainty of heat flux density was estimated as follows:(3)

Where the absolute measurement errors of the electrical power Δ*P*_max_, outside tube diameter Δ*D*_o _and active length of a tube Δ*L *are 10 W, 0.02 mm, and 0.2 mm, respectively. So, the maximum overall experimental limits of error for heat flux density extended from ± 1.3% for maximum heat flux density up to ± 1.2% for minimum heat flux density.

The experimental uncertainty for the average heat transfer coefficient is calculated as(4)

where the absolute measurement error of the wall superheat, δT, estimated from the systematic error analysis equals ± 0.2 K. The maximum error for average heat transfer coefficient was estimated to ± 2.3%.

## Results

Investigation of nucleate saturated pool boiling heat transfer on the outside of smooth horizontal tubes submerged in water-Al_2_O_3 _and water-Cu nanofluids has been carried out. The measurements were performed at atmospheric pressure and nanoparticles concentration of 0.01%, 0.1%, and 1% by weight.

### Comparison of present results with literature data

**Figure 10:**
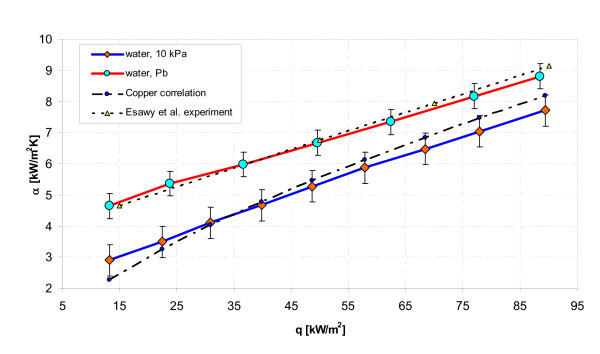
**Variation of present results with Cooper correlation **[25]**and Esawy et al**. experimental data [26].

In order to validate the apparatus as well as experimental procedure, the present data for distilled water at subatmospheric pressure were compared with those predicted by Cooper correlation [25](1A)

and present results for atmospheric pressure have been compared with the experimental data by Esawy et al. [26] recorded for distilled water boiling on a smooth horizontal stainless steel tube of almost the same diameter (12.7 mm) heated by a cartridge heater. Figure 10 shows comparison of present experimental data with Cooper correlation taking heat flux density as abscissa and heat transfer coefficient as ordinate for the pool boiling of distilled water on horizontal stainless steel smooth tube. The experimental data for the boiling of distilled water are found to be in reasonable agreement with those predicted by Cooper correlation (within a band error ± 4.5%) as well as those obtained experimentally by Esawy et al. (within a band error ± 2%).

### Effect of nanoparticle material

**Figure 11:**
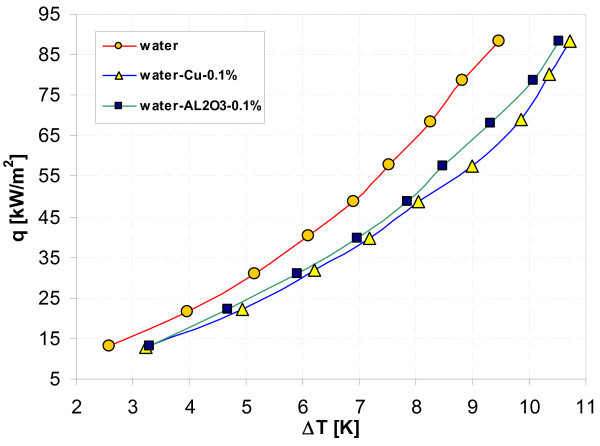
**Boiling curves of smooth copper tube in water-Al_2_O_3 _and water-Cu nanofluids with 0.1% concentration**.

**Figure 12:**
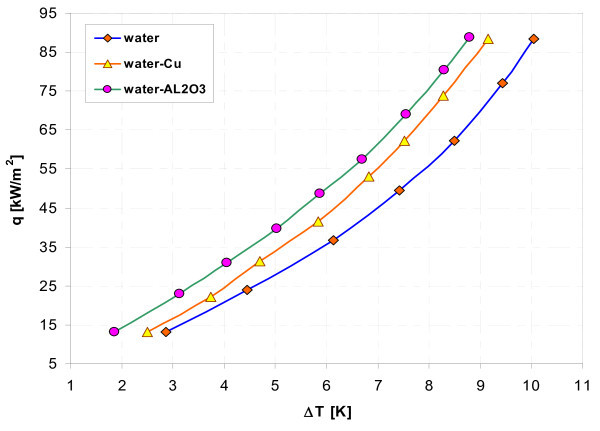
**Boiling curves of smooth stainless steel tube in water-Al_2_O_3 _and water-Cu nanofluids with 0.1% concentration**.

**Figure 13:**
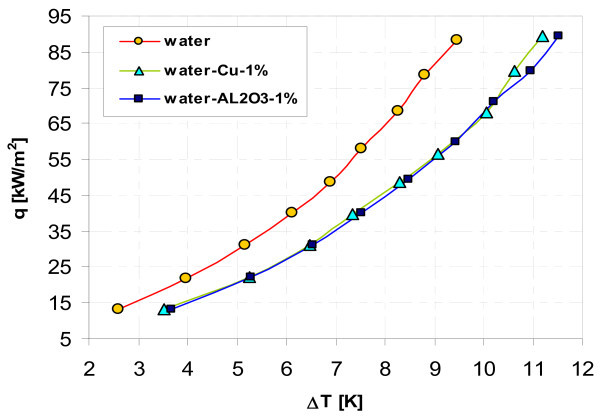
**Boiling curves of smooth copper tube in water-Al_2_O_3 _and water-Cu nanofluids with 1% concentration**.

**Figure 14:**
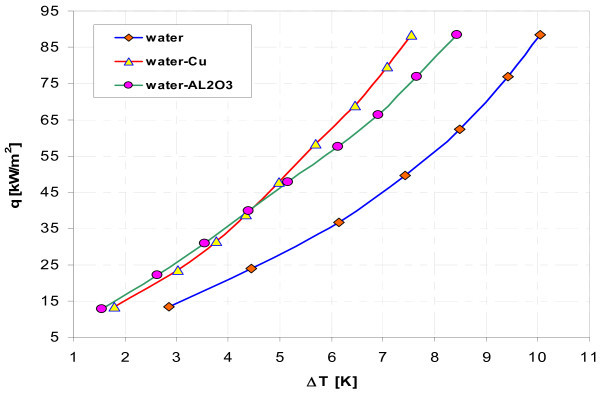
**Boiling curves of smooth stainless steel tube in water-Al_2_O_3 _and water-Cu nanofluids with 1% concentration**.

Figures 11, 12, 13, and, 14 display influence of nanoparticle material on heat transfer during boiling of water-Al_2_O_3 _and water-Cu nanofluids on smooth copper and stainless steel tubes. Independent of concentration (0.1% and 1%), nanoparticle material (Al_2_O_3 _and Cu) has almost no influence when boiling of nanofluid water-Al_2_O_3 _or water-Cu takes place on smooth copper tube - Figures 11 and 13. Moreover, the adding of nanoparticles degrades heat transfer performance while boiling of water-Al_2_O_3 _and water-Cu nanofluids on copper smooth tube. For stainless steel tube and lower concentration tested (0.1%) - Figure 12, higher heat flux density was obtained for water-Al_2_O_3 _nanofluid than for water-Cu nanofluid. Water-Cu nanofluid displays slight superiority over water-Al_2_O_3 _nanofluid - with the same 1% concentration of nanoparticles, while boiling on smooth stainless steel tube and heat flux density above 40 kW/m^2 ^- Figure 14.

### Effect of nanofluid concentration

**Figure 15:**
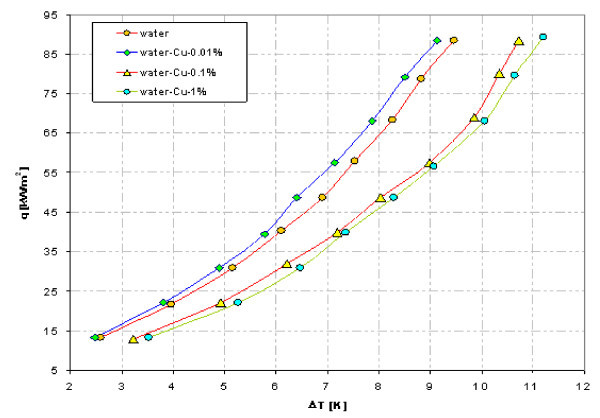
**Boiling curves of copper tube in water-Cu nanofluid**.

**Figure 16:**
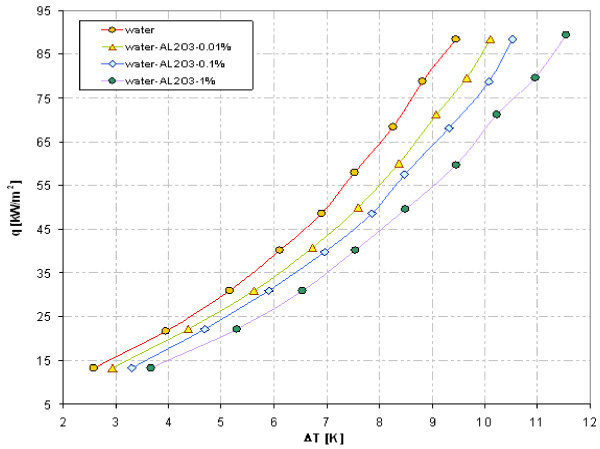
**Boiling curves of copper tube in water-Al_2_O_3 _nanofluid**.

As an example, Figures 15 and 16 illustrate influence of nanoparticle concentration on heat transfer during boiling on smooth copper tube. Contrary to stainless steel tube experiments - Figures 12 and 14, the adding of copper as well as Al_2_O_3 _nanoparticles deteriorates pool boiling heat transfer, shifting the boiling curve to the right. The higher concentration of nanoparticles was the lower heat transfer coefficient got for the same wall superheat.

### Effect of tube material

**Figure 17:**
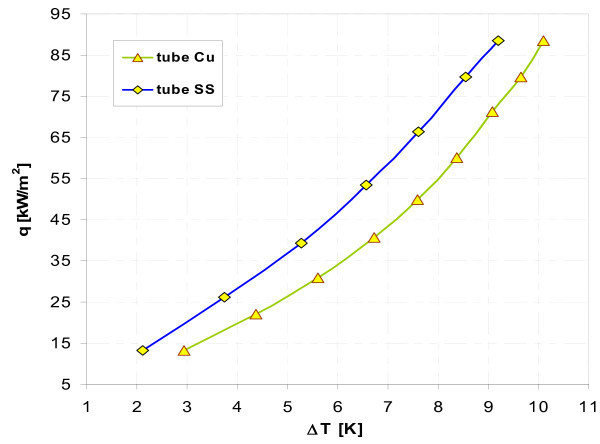
**Boiling curves of water-Al_2_O_3 _(0.01%) nanofluid**.

**Figure 18:**
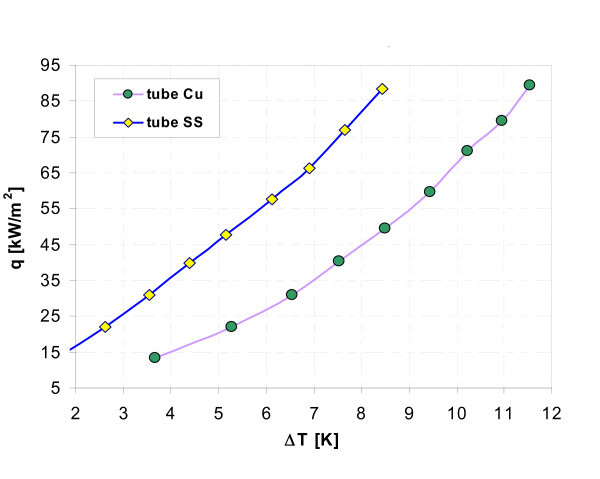
**Boiling curves of water-Al_2_O_3 _(1%) nanofluid**.

**Figure 19:**
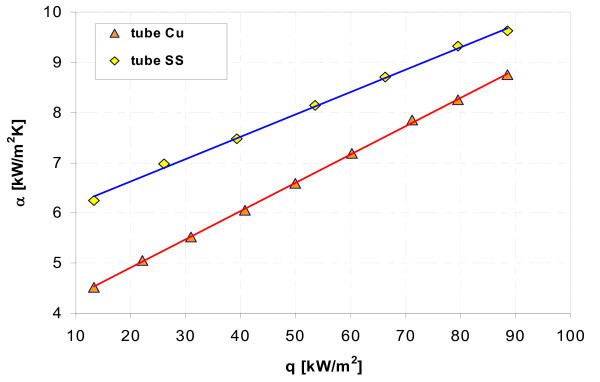
**Heat transfer coefficient during boiling of water-Al_2_O_3 _(0.01%) nanofluid**.

**Figure 20:**
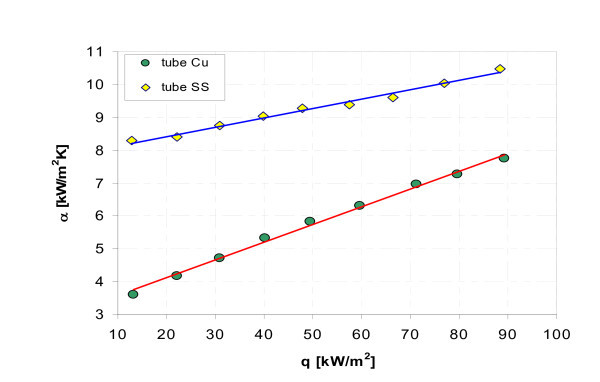
**Heat transfer coefficient during boiling of water-Al_2_O_3 _(1%) nanofluid**.

**Figure 21:**
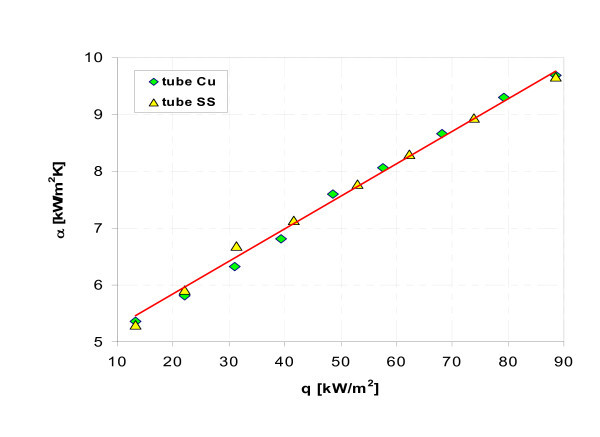
**Heat transfer coefficient during boiling of water-Cu (0.1%) nanofluid**.

**Figure 22:**
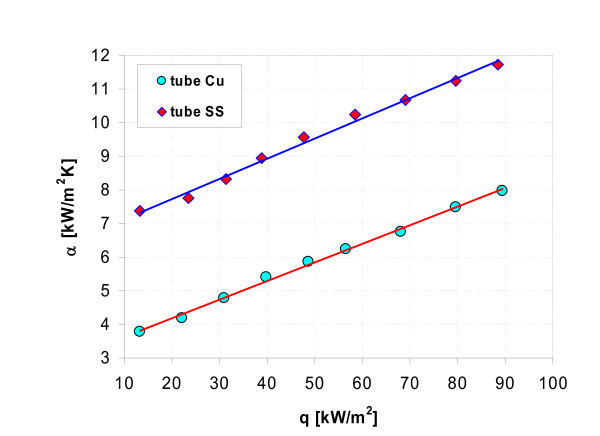
**Heat transfer coefficient during boiling of water-Cu (1%) nanofluid**.

Figures 17, 18, 19, 20, 21, and 22 show influence of heating surface material (tube material) on heat transfer while boiling of water-Al_2_O_3 _or water-Cu nanofluids of different concentrations. Figures 17 and 18 illustrate boiling curves of smooth stainless steel and copper tubes in water-Al_2_O_3 _nanofluid with 0.01% and 1% nanoparticle concentrations, respectively. Independent of concentration (0.01% and 1%) distinctly higher heat transfer coefficient was recorded for stainless steel tube - Figures 19 and 20, respectively. Figures 21 and 22 illustrate heat transfer coefficient against heat flux density for smooth stainless steel and copper tubes in water-Cu nanofluid with 0.1% and 1% nanoparticle concentrations, respectively. It seems that surface material does not affect the boiling heat transfer in 0.1% water-Cu nanofluid - Figure 21, but for 1% nanoparticle concentration, again like for water-Al_2_O_3 _nanofluid (Figure 17) distinctly higher heat transfer coefficient was recorded for stainless steel tube - Figure 22.

## Conclusions

Independent of the concentrations tested (0.01%, 0.1%, and 1% by weight) nanoparticle material (Al_2_O_3 _and Cu) has almost no influence while boiling of water-Al_2_O_3 _or water-Cu nanofluids on smooth copper tube.

Contrary to stainless steel tube experiments, the adding of copper as well as Al_2_O_3 _nanoparticles deteriorates pool boiling heat transfer on copper smooth tubes. The higher concentration of nanoparticles was the lower heat transfer coefficient got for the same wall superheat.

Independent of concentration distinctly higher heat transfer coefficient was recorded for stainless steel tube than for copper tube for the same heat flux density. It seems that surface material does not affect the boiling heat transfer in 0.1% water-Cu nanofluid.

A thin solid coating (detected by eye) was observed on copper tubes after tests with water-Al_2_O_3 _and water-Cu nanofluids. The higher concentration of the nanoparticles was the thicker coating was recorded at the end of testing.

## Competing interests

The authors declare that they have no competing interests.

## Authors' contributions

JTC conceived of the study, carried out the literature review, drafted the manuscript and participated in the design and coordination of the study and discussion of the results. TZK conducted the experiments, prepared all figures and participated in the discussion of the results. Both authors read and approved the final manuscript.
